# The urgent need to cut methane emissions

**DOI:** 10.1093/nsr/nwab221

**Published:** 2021-12-04

**Authors:** Euan G Nisbet

**Affiliations:** Department of Earth Sciences, Royal Holloway University of London, UK

**Figure 1.**
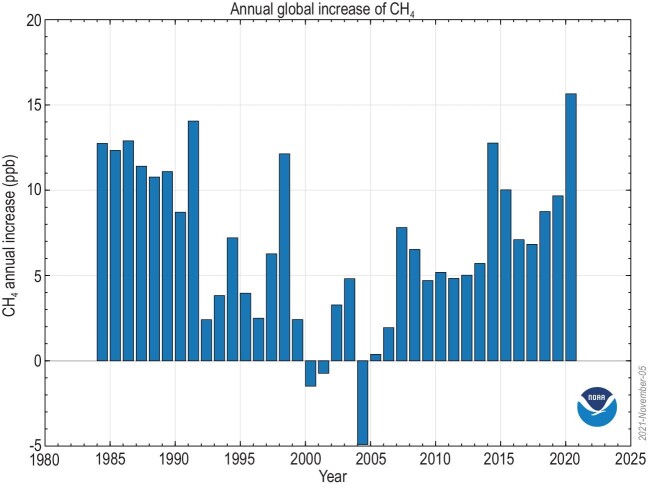
Annual global increase in atmospheric methane (parts per billion). Plot from the US National Oceanic and Atmospheric Administration (NOAA) trends in CH_4_https://gml.noaa.gov/ccgg/trends_ch4/.

Atmospheric methane is a powerful greenhouse gas, surpassed only by CO_2_ in its human-added warming impact. From the start of industrialization, when humans began altering the air, the amount of methane in the atmosphere has grown proportionately more than CO_2_. In 2020, methane's growth was the highest in the detailed observational record (Fig. 1). But although there has been much work on the global methane budget [1], we do not fully understand why this recent growth is taking place, nor why growth seems to be increasingly strong [2,3]. Is methane's rise driven by new human emissions? Are feedbacks driving increased natural emissions as warming feeds warming? Or is something happening to reduce the oxidative power of the atmosphere and thus weaken the main methane sink, destruction by atmospheric hydroxyl, OH?

In this issue, Zhang *et al.* [4] investigate the causes of methane growth by using both source estimates and isotopic source signatures. Carbon has two stable isotopes—common carbon-12 and less common carbon-13. Methane sources have distinctive carbon isotopic signatures, expressed as δ^13^C_CH4_, which is a measure of comparison against a carbonate standard. Methane's recent growth post-2007 has been marked by a sustained shift to more negative δ^13^C_CH4_ [3]. Biogenic methane, from wetlands, cattle, landfills, etc., is relatively poor in carbon-13 (i.e. very negative δ^13^C_CH4_), while thermogenic methane, from geological sources and also produced in fires, tends to be relatively richer in carbon-13 (i.e. less negative δ^13^C_CH4_).

Hitherto, most inventory assessments of methane budgets have not been isotopically balanced or tested for compatibility with isotopic signature information. By using these powerful isotopic constraints on the budget, Zhang *et al.* [4] conclude that the main factor driving methane's growth since 2007 is human activity—increased emissions from agriculture, landfills and waste, as well as from fossil fuels. These conclusions may be challenged as tropical sources are still very poorly studied isotopically, and some of methane's growth may be driven by feedback impacts (warming feeding warming), especially on natural wetlands. Nevertheless, this study by Zhang *et al.* [4] is important and path-breaking, demonstrating that future global, regional and national methane emission inventories will need to make full use of isotopic information.

Recently, the United Nations issued its Global Methane Assessment [5] and Emissions Gap Report [6], urging action on methane, which has led to the Global Methane Pledge [7]. Mitigating methane emissions is widely feasible and often inexpensive [8]. For example, landfills are major sources, easily mitigated by gas extraction or simply by soil cover. Leaks from gas pipes and boilers can be stopped. Methane can be removed from high-methane air from coal mine vents, and around cow manure and pig slurry tanks. Crop waste fires are a substantial methane source, and also a major cause of air pollution: emissions can be cut by burning crop wastes in cleaner power-generating incinerators. Much can be done, and done cheaply. The results of Zhang *et al.* [4] challenge every nation to act, and to act now.

***Conflict of interest statement*.** None declared.
